# Analysis of the clinical characteristics of five patients with Kawasaki disease complicated with macrophage activation syndrome in Baoding Hospital of Beijing Children’s Hospital, Capital Medical University

**DOI:** 10.12669/pjms.41.5.11538

**Published:** 2025-05

**Authors:** Wenjing Zhang, Huiqing Feng, Jing Zhao, Man Li, Yanping Liu

**Affiliations:** 1 Wenjing Zhang, Department of Cardiology, Baoding Hospital of Beijing Children’s Hospital, Capital Medical University, 3399, Hengxiang North Street, Baoding, Hebei Province 071000, China; 2 Huiqing Feng, Department of Cardiology, Baoding Hospital of Beijing Children’s Hospital, Capital Medical University, 3399, Hengxiang North Street, Baoding, Hebei Province 071000, China; 3 Jing Zhao, Department of Cardiology, Baoding Hospital of Beijing Children’s Hospital, Capital Medical University, 3399, Hengxiang North Street, Baoding, Hebei Province 071000, China; 4 Man Li, Department of Cardiology, Baoding Hospital of Beijing Children’s Hospital, Capital Medical University, 3399, Hengxiang North Street, Baoding, Hebei Province 071000, China; 5 Yanping Liu, Department of Cardiology, Baoding Hospital of Beijing Children’s Hospital, Capital Medical University, 3399, Hengxiang North Street, Baoding, Hebei Province 071000, China

**Keywords:** Kawasaki disease, Macrophage activation syndrome

## Abstract

**Objective:**

To investigate clinical characteristics of Kawasaki disease (KD) complicated with macrophage activation syndrome (MAS) and to identify possible indicators for early diagnosis.

**Method:**

Clinical data, laboratory indexes, treatment, and prognosis of five patients with KD complicated with MAS admitted to Department of Cardiology, Baoding Children’s Hospital from April 2020 to April 2022 were retrospectively analyzed.

**Results:**

Five pediatric patients aged between three months and five years were included in the study. The enrolled patients were non-reactive to intravenous immunoglobulin (IVIG) and presented with mental impairment, abdominal distension, skin rash, and scleroderma of the hands and feet. Four patients had hepatomegaly and two patients had splenomegaly. There were four patients with coronary artery diseases. Triglyceride (TG) levels were increased or showed an upward trend in four patients. Two patients had positive bone marrow hemophagocytosis. All patients had decreased fibrinogen, increased ferritin levels, a decrease or a downward trend in the natural killer (NK) lymphocytes counts, and significantly increased sCD25. Four patients presented with decreased activity of NK cells. All pediatric patients enrolled were treated with two doses of high-dose IVIG +methylprednisolone. One patient died as a result of stopping the treatment, while four remaining patients had a good prognosis.

**Conclusions:**

MAS complication should be suspected in pediatric patients with KD who are non-responsive to IVIG and show signs of mental impairment, abdominal distension, scleroderma of hands and feet, or hepatosplenomegaly. We should monitor trends in blood routine, ferritin, triglyceride, fibrinogen and NK lymphocytes counts.

## INTRODUCTION

Kawasaki disease (KD) is an acute systemic vasculitis that impacts small- and medium-size blood vessels and carries with it the risk of coronary artery aneurysm.^1^,^2^ However, most children will only present with acute symptoms without cardiac complications. While KD is a rare disorder, it is considered one of the most common vascular inflammatory disorders of childhood together with Henoch Schonlein, and is characterized by an early onset.^3^ Approximately 85% of children with KD are younger than five years of age, and the age of onset peaks at 6-24 months. While the disease is uncommon in children younger than five years of age, if the onset occurs at this age, the risk of coronary artery aneurysm is increased, and boys are more susceptible to KD than girls.^1^-^4^ The etiology of KD is unknown. Studies suggest that in some genetically susceptible individuals, the disease is triggered by infection that may promote organismal hypersensitivity or an abnormal immune response that leads to inflammation and damage of the vessel wall.^1^,^2^,^5^

Macrophage activation syndrome (MAS), a severe complication of systemic inflammatory diseases in children, is mainly reported in systemic-onset juvenile idiopathic arthritis (sJIA) and rarely in KD. Moreover, it is a particular secondary hemophagocytic lymphohistiocytosis (HLH), described as rheumatoid immunity-related HLH. A small number of patients with KD complicated with MAS have been reported earlier, with an incidence of 1.1%-1.9% and a mortality rate of 25%.^3^,^5^-^7^ Therefore, early identification of MAS complication of KD and timely treatment are crucial to control inflammation quickly and avoid deterioration. In this study, we retrospectively investigated five pediatric patients with KD-MAS admitted to our hospital to identify the clinical and lab indicators for early detection and diagnosis of KD-MAS.

## METHODS

Five pediatric patients with KD complicated with MAS who were hospitalized in the Department of Cardiology, Baoding Children’s Hospital from April 2020 to April 2022 were selected for the study. Children met the diagnostic criteria of KD based on the KD AHA statement 2017^5^,^6^ or JCS 2020^7^ guidelines and the diagnostic criteria of HLH^8^ based on the HLH-2004 guideline.

### Ethical approval:

This study was approved by the ethics committee of Baoding Children’s Hospital (No. 2022-05, Date: August 12, 2023) and informed consent were obtained.

### Data collection:

Clinical and laboratory characteristics of five patients with KD complicated with MAS were collected during the acute phase, characterized by rash, cervical lymphadenopathy (> 1.5 cm in diameter), bilateral bulbar conjunctival hyperemia (noninfectious), orolabial changes (dry red and fissured lips, myrica tongue), swelling or finger end desquamation of hands and feet, perianal desquamation, and total fever process.

### Laboratory indicators for the different stages of the fever process were collected:

Platelets (PLT) total white blood cell (WBC), hemoglobin (HGB), erythrocyte sedimentation rate (ESR), albumin (ALB), alanine transaminase (ALT), C-reactive protein (CRP); blood phages; natural killer (NK) lymphocyte count; alpha chain of the soluble interleukin-2 receptor (sCD25); and acute phase echocardiographic results. Coronary artery injury was diagnosed according to the Practical Pediatrics diagnostic criteria for coronary artery injury: 1) Coronary artery dilation: ≥ 2.5 mm internal diameter of coronary artery in those ≤ 3 years old; > Age 3-9 years, coronary artery internal diameter ≥ 3.0 mm; > 9-14 years old with coronary artery diameter ≥ 3.5 mm; 2) Coronary aneurysm: coronary artery internal diameter > 4 mm.

## RESULTS

Five pediatric patients (four boys, 80%; one girl, 20%) with KD complicated with MAS were enrolled. The onset age of the patients ranged from 3 months to 5 years old, with a mean of 1.83±1.53 years. All the enrolled patients were not comorbid with any other underlying diseases (Table-I).

**Table-I:** Clinical information of the five KD-MAS pediatric patients enrolled.

Case	Gender	Fever process (days)	Fever	Skin rash	Hepatomegaly	Splenomegaly	Cervical lymphadenopathy	Abdominal distention	Mental weakness	Scleroderma of hands and feet	coronary artery disease	IVIG unresponsive
1	Male	20	+	+	+	+	-	+	+	+	+	+
2	Female	10	+	+	+	-	-	+	+	+	+	+
3	Male	17	+	+	-	-	-	+	+	+	-	+
4	Male	17	+	+	+	-	+	+	+	+	+	+
5	Male	28	+	+	+	+	-	+	+	+	+	+

### Clinical symptoms and signs:

All five pediatric patients developed a persistent fever, with a maximum temperature of 39.5-40.2°C that lasted for 10-25 days, with an average fever process of 18.4±6.5 days. All patients had skin rashes, abdominal distension, and mental impairment. Four (80%) and two (40%) patients had hepatomegaly and splenomegaly, respectively. One patient (20%) had cervical lymphadenopathy, and four (80%) had coronary artery diseases (Table-I). None of patients was reactive to intravenous immunoglobulin (IVIG). One patient had both KD shock syndrome and KD-MAS. Four patients were diagnosed with complete KD and one was diagnosed with incomplete KD. MAS onset happened in the acute phase in four cases and the subacute phase in one case.

### Laboratory and auxiliary examination:

### General lab indicator:

As shown in Table-II, progressive decrease in hemoglobin and platelet levels was found in all patients. Patients No.5 and No.4 showed increased ALT and AST, respectively. Three patients had a normal ESR, and two had elevated ESR, which gradually decreased. TG levels increased or showed an upward trend in four cases. Bone marrow hemophagocytosis was positive in two cases. All patients had decreased fibrinogen levels, increased ferritin levels, and decreased or trending lower NK counts. Four cases showed decreased activity of NK cells, and all patients had significantly increased sCD25 (Table-III). All five enrolled patients underwent lumbar puncture, and no abnormality was found in the cerebrospinal fluid. Four patients (80%) had coronary artery diseases, with coronary artery dilatation. Three patients had left coronary artery dilatation, and one had right coronary artery dilatation.

**Table-II:** Lab indicator of the five KD-MAS pediatric patients enrolled.

Case No.	Fever (day*)	WBC (× 10^9^/L)	RBC (× 10^12^/L)	HGB (g/L)	PLT (× 10^12^/L)	ESR (mm/h)	Fid (g/L)	ferritin (ng/mL)	TG (mmoL/L)	AST (U/L)	ALT (U/L)	NK (U/mL)
1	1	9.64	3.45	96	215	10	3.32	3.0	0.57	128	32	1281
	5	21.49	2.68	74	199	9				31	14	
	10	38.81	2.35	65	82	5	1.72	340	3.21	33	11	104
2	7	13.28	4.14	116	107	18	3.34	1506	6.08	55	51	63
	8	9.87	3.46	99	103	15	2.58	1670	3.73	60	59	58
3	3	4.91	4.23	117	136	14	2.39	340	1.21	60	23	
	8	8.59	4.11	111	124	15	1.93	1872	2.02	62	20	6
	11	8.15	3.22	92	119	15	1.91	1945	2.25	26	18	
4	2	8.86	5.03	116	252	90	5.92	104	1.3	32	60	259
	9	17.46	4.16	95	76	88	3.18	124	2.11	13	16	
	10	17.09	3.64	85	108	85	2.95	152	2.33	21	16	
	13	39.94	3.28	76	363	85	1.77	245	3.45	15	8	142
	14	43.53	3.11	73	605	80	1.44	287	4.17	19	8	24
5	8	10.64	4.67	129	454	90	4.59	124	1.37	17	25	262
	14	7.44	4.29	115	332	60	3.29	234	1.38			
	17	3.97	4.10	110	164	55	2.95	756	1.30			
	18	2.92	3.67	99	146	50	2.84	851	1.4	363	204	102

**Table-III:** Lab indicator of the five KD-MAS pediatric patients enrolled.

Case No.	Blood phages	NK cytoactive (%)	sCD25 (U/ml)
1	+	3.2	15320
2	+	3.5	15610
3	-	3.0	12360
4	-	1.1	8370
5	-	22.48	23240

***Note:*** NK cytoactive: normal reference range≥ 3.0%; sCD25: normal reference range:223-710 U/ml.

### Disease progression, treatment, and prognosis of the pediatric patients enrolled:

All five pediatric patients were unresponsive to the first dose of IVIG and were treated with gamma globulin (2 g/kg) + methylprednisolone (5 mg/kg) as soon as showed the trend towards MAS complication without waiting for the definite diagnosis. One patient was also supplemented with cyclosporine A, but eventually died because their parents gave up the treatment. The prognosis of the remaining four patients was good.

## DISCUSSION

The cases of KD combined with MAS are very scarce, with an incidence rate of 1.1% to 1.9%, especially in children over five years old.^9^ In this study, the overall age of patients with KD combined with MAS was relatively young, ranging from three months to five years. Moreover, the average age of onset was 1.83±1.5 years, which was consistent with the peak age of KD. Our study included four boys (80%) and one girl (20%), and the gender ratio (4:1 in males: females) was higher than that reported by Garcia et al.^5^ (about 68%), which might be related to the small sample size of this study. MAS may develop at any stage of KD, including acute, subacute, and recovery stages. While MAS may be diagnosed sooner than KD, these two conditions usually appear together.^9^ In this study, four patients acquired MAS during the acute phase and one patient was diagnosed with MAS during the subacute phase, suggesting that monitoring at any time is necessary to prevent misinterpretation and missed diagnosis.

The clinical manifestations are generally unspecific in patients with KD complicated with MAS. In this study, the fever process of all five pediatric patients was ≥ 10 days, and all patients presented with mental impairment, abdominal distension, rash, and scleroderma of hands and feet. Moreover, skin rashes did not disappear easily or recur. The above-mentioned indicators may be considered the characteristics of KD complicated with MAS to help in early recognition. While hepatosplenomegaly is relatively rare in ordinary KD patients, the incidence of hepatosplenomegaly rises to 69% in patients with KD complicated with MAS.^5^

In our study, 80% and 40% of patients developed hepatomegaly and splenomegaly, respectively. Our results suggest that there is a need to increase the awareness of possible MAS complications in pediatric KD patients with signs of hepatosplenomegaly. Previous studies have demonstrated that the incidence rate of coronary artery disease in pediatric patients with KD without treatment is 15%-30%. This incidence rises to 46% in pediatric patients with KD complicated with MAS,^5^ indicating that pediatric KD patients with coronary artery injury are at a higher risk of KD-MAS. In our study, the incidence of coronary artery disease was as high as 80%, which was higher than in previous reports, which might be related to the small sample size of this study.

Our results strongly suggest that clinicians should pay close attention to the presence of coronary artery disease during the diagnosis and treatment of this population of patients. Current studies reported that pediatric patients with KD who were intolerant to IVIG and had a long fever process were more likely to develop MAS.^10^,^11^ Some studies indicated that non-reactivity to IVIG of KD patients was one of the risk factors for MAS complications.^1^,^12^,^13^ In this study, all pediatric patient were not reactive to IVIG, consistent with previous reports. Considering high mortality rates in patients with KD complicated with MAS, it is necessary to be highly vigilant about the possibility of this condition when pediatric patients with KD show no response to the first dose of IVIG treatment and are accompanied by mental impairment, abdominal distension, skin rashes that do not disappear easily, scleroderma of hands and feet, or hepatosplenomegaly.

To date, neither an international HLH diagnostic standard nor a specific diagnostic standard of KD complicated with MAS has been established. In most cases, the diagnostic criteria formulated by the international organization cell association in 2004 have been used; that is, HLH diagnosis can be established by satisfying one of the following two criteria: (a) consistent with the molecular diagnosis of HLH: PRF1, UNC13D, Munc18-2, Rab27a, STX11, AH2D1A or BIRC4 gene mutations; (b) meeting five of the following eight diagnostic criteria: 1) fever; 2) splenomegaly; 3) hemocytopenia (affecting two or three peripheral blood cell lines): hemoglobin <90 g/L (newborn: hemoglobin <100 g/L), platelet < 00×109/L, neutrophils <1.0×109/L; 4) hypertriglyceridemia and/or hypofibrinogenemia: fasting triglyceride ≥3.0 mmol/L (2.65 g/L), fibrinogen 1.5 g/L; 5) hemophagocytosis rather than malignant transformation in bone marrow, spleen or lymph nodes; 6) decreased or deficient NK cytoactive; 7) ferritin ≥500 μg/L; 8) soluble CD25 (sIL-2R) ≥2400 U/mL.All five pediatric patients enrolled in this study met these diagnostic criteria, although clinically, the sensitivity of these diagnostic criteria for KD-MAS is quite low.^1^ Gene detection was performed for all five enrolled pediatric patients. While on the one hand, gene detection took a long time on the other, it was previously reported that HLH-related pathogenic gene mutations are not found in pediatric patients with KD-MAS. However, many hospitals are not capable of detecting the activity of NK cells and level of soluble CD25, and the external examination takes a long time. Hence, detection of genes involved in the activity of NK cells and level of soluble CD25 was not suitable for the early diagnosis of KD-MAS.García-Pavón et al.^5^ reported that the detection rate of hemophagocytosis was as high as 88% in bone marrow, lymph nodes, liver, and spleen of patients with KD complicated with MAS.^5^ Some studies have reported a positive rate of biopsy of only 40%-50%.^12^,^13^ In this study, the detection rate of hemophagocytosis in bone marrow was 40%, suggesting that MAS could not be ruled out even if hemophagocytes were not found in the biopsy.

Additionally, as biopsy is an invasive procedure, it is not suitable for early examination. Therefore, it is imperative to find other laboratory indicators to help early detection and diagnosis of KD-MAS. Elevated ferritin in serum, liver function damage, and abnormal coagulation function are characteristic laboratory manifestations of MAS.^14^ In this study, three patients had ferritin ≥500 μg/L, suggesting that not all cases of KD complicated with MAS exhibits significantly increased level of ferritin. Cron et al.^15^ also found that MAS may be present in an individual with relatively low ferritin levels. However, all five pediatric patients in the current study showed an increasing trend of ferritin, suggesting this might be more clinically significant. Additionally, three KD+MAS patients in our study had elevated ALT, and four had elevated AST, consistent with the conclusion in previous studies that hyperferriemia and elevated AST are indicative of KD complicated with MAS.^9^

Our results also indicated that fibrinogen and TG levels, as monitoring indicators for MAS diagnosis, also show a certain changing trend in KD+MAS patients. Levels of fibrinogen decreased, and TG increased in four patients. Another indicator, ESR, is affected by plasma components. Due to hypofibrinogenemia, ESR in pediatric patients with MAS decreases to a certain extent, while ESR in ordinary pediatric patients with KD usually increases. In this study, three patients had normal ESR, and two had elevated ESR, but showed a downward trend. All five pediatric patients also showed a decrease or a downward trend in the NK lymphocyte count. The decrease in NK lymphocyte count is common in children with infection, advanced malignant tumors, and severe diseases combined with immunodeficiency. Whether it can be used as a predictor of KD complicated with MAS needs a large sample study. The changes in the above laboratory indicators were found 3-4 days earlier than the definite diagnosis of KD complicated with MAS. The expert consensus on the diagnosis and acute treatment of KD^9^ indicates that the diagnostic standard of KD complicated with MAS should be lower than that of rheumatoid immunity-related hemophagocytic lymphohistiocytosis. Based on our results, we suggest that MAS complication should be suspected if ferritin in serum in pediatric patients with KD increases progressively, combined with two or more of the following:

- A sharp decrease in platelet count.
- Several times higher level of aspartate aminotransferase than baseline.
- Sharp increase in TG.
- Significant decrease in fibrinogen.
- Hemophagocytes are found in bone marrow or other tissues (e.g., lymph node, liver, and spleen). Our results further emphasize that the trend of laboratory indicators is more conducive to the early detection of KD complicated with MAS.

To date, there is no unified treatment for MAS, and glucocorticoids are still the first-line treatment for KD-MAS.^16^,^17^ It has been reported that timely administration of intravenous glucocorticoids could reach a 50% cure rate.^18^ Lin et al.^19^ proposed that glucocorticoids should be used early, and the course of treatment should be appropriately extended in pediatric patients with KD complicated with MAS. Furthermore, when the symptoms cannot be controlled by glucocorticoids, pediatric patients should be supplemented with biological agents or other immunosuppressants. The five pediatric patients enrolled were treated with high-dose IVIG (2g/kg) combined with intravenous glucocorticoid when we found the trend of KD combined with MAS, and then the drugs were gradually reduced and stopped, with a total treatment course of eight weeks. Four patients were clinically cured, and one patient died due to discontinuation of treatment. In summary, glucocorticoids should be used as soon as possible for pediatric patients with KD-MAS once they show a trend of MAS development, which might improve the remission rate of the disease.

### Strengths and Limitations:

This study emphasizes the changing trends of indicators, which can detect Kawasaki disease with MAS earlier than previous reports, and provide early and timely treatment, greatly improving the prognosis of the children. However, due to the low incidence of KD with MAS, the sample size of this study is small, which may affect the experimental results. We will extend the time for collecting medical records and try to enlarge the sample size to make the experimental results more convincing.

## CONCLUSIONS

Pediatric patients with KD should be monitored closely if they do not react to the first dose of IVIG. In young patients with KD who have mental impairment, abdominal distension, skin rashes that do not go away easily, scleroderma of the hands and feet, or hepatosplenomegaly, clinicians should be particularly cautious of the potential MAS complication. Changes in regular blood tests, ferritin, TG, fibrinogen, ESR, and NK lymphocyte count should be monitored on a continuous basis. For pediatric patients with KD who show a progressive decline in one or two lines of routine blood tests, there is a need to perform additional monitoring of indexes to detect an increase in ferritin, a decline in ESR, a progressive increase in TG, a progressive decline in fibrinogen, or a progressive decline in NK lymphocyte count. Bone marrow puncture, cytoactive NK, and soluble CD25 detection should be done. Furthermore, early treatment of the second high-dose gamma globulin paired with glucocorticoid may significantly improve the prognosis of children with KD complicated by MAS.

### Authors’ contributions:

**WZ:** Study design, literature search and manuscript writing.

**HF, JZ, ML and YL:** Were involved in data collection, data analysis and interpretation. Critical Review.

**WZ:** Was involved in the manuscript revision and validation.

All authors have read and approved the final manuscript and are accountable for the integrity of the study.
